# Role of RGM coreceptors in bone morphogenetic protein signaling

**DOI:** 10.1186/1750-2187-2-4

**Published:** 2007-07-05

**Authors:** Peter J Halbrooks, Ru Ding, John M Wozney, Gerard Bain

**Affiliations:** 1 Quality Control Technical Services Laboratory, Genzyme Corporation, Framingham MA, 01701, USA; 2 Women's Health and Musculoskeletal Biology, Wyeth Discovery Research, Cambridge, MA, 02140, USA

## Abstract

**Background:**

The repulsive guidance molecule (RGM) proteins, originally discovered for their roles in neuronal development, have been recently identified as co-receptors in the bone morphogenetic protein (BMP) signaling pathway. BMPs are members of the TGFβ superfamily of signaling cytokines, and serve to regulate many aspects of cellular growth and differentiation.

**Results:**

Here, we investigate whether RGMa, RGMb, and RGMc play required roles in BMP and TGFβ signaling in the mouse myoblast C2C12 cell line. These cells are responsive to BMPs and are frequently used to study BMP/TGFβ signaling pathways. Using siRNA reagents to specifically knock down each RGM protein, we show that the RGM co-receptors are required for significant BMP signaling as reported by two cell-based BMP activity assays: endogenous alkaline phosphatase activity and a luciferase-based BMP reporter assay. Similar cell-based assays using a TGFβ-induced luciferase reporter show that the RGM co-receptors are not required for TGFβ signaling. The binding interaction of each RGM co-receptor to each of BMP2 and BMP12 is observed and quantified, and equilibrium dissociation constants in the low nanomolar range are reported.

**Conclusion:**

Our results demonstrate that the RGMs play a significant role in BMP signaling and reveal that these molecules cannot functionally compensate for one another.

## Background

The transforming growth factor β (TGFβ) superfamily of ligands, which includes the TGFβ proteins, bone morphogenetic proteins (BMPs), growth and differentiation factors (GDFs), and others, plays a key role in regulating cell proliferation, differentiation, migration, and apoptosis in a diverse set of developmental and physiological pathways. These secreted signaling proteins exert crucial functions at several stages of development and are essential for tissue repair and maintenance in the adult [1-4]. The pathogenesis in a host of diseases, including cancer, osteoporosis, and fibrosis, has been attributed to disregulated TGFβ superfamily function [5]. The BMPs, the largest subgroup within the TGFβ superfamily, participate in the development of nearly all organs and play a critical role in the formation and repair of bone [6,7]. The TGFβ s are known to play important roles in cellular proliferation and differentiation, inflammation and tissue repair, and host immunity [4,8,9].

TGFβ family ligands mediate their effects by binding to specific pairs of membrane bound type I and type II serine/threonine kinase receptors, leading to the activation of distinct intracellular Smad pathways. These phosphorylated receptor-regulated Smads are translocated from the cytoplasm to the nucleus where they function to assist in specific gene activation. In general, ligands in the TGFβ superfamily activate either of two Smad branches. The BMPs activate Smads 1/5/8, while the TGFβ/Activins activate Smads 2/3, although recent reports have demonstrated that there can be rare exceptions to this rule [10].

A multitude of regulatory mechanisms are required for these functions, in order to ensure that this powerful network of signaling molecules functions with specific distribution and activation. Such mechanisms include ligand-specific extracellular antagonists [7], molecular recognition of the type I and type II cellular receptors [11], and the presence of cell surface pseudoreceptors [12]. An emerging, yet important, regulatory mechanism for some TGFβ superfamily members is the presence of co-receptors (also called accessory or type III receptors) which promote or inhibit ligand binding [13-15]. Two examples are that of betaglycan, which plays an essential role in TGFβ signaling [16], and endoglin, which has been implicated in Alk1 ligand signaling [17]. An additional co-receptor, cripto, has been shown as an essential cofactor in Nodal signaling [18]. Co-receptors do not appear to have any intrinsic signaling activity, but rather serve to regulate TGFβ access to the signaling receptors [4].

Recently, three members of the repulsive guidance molecule (RGM) family have been implicated in the BMP signaling pathway [19-22]. RGMa, RGMb (DRAGON), and RGMc (hemojuvelin/HFE2) have all been shown to enhance cellular responses to BMP signals, and also to bind BMP2 ligand. In addition to sharing significant sequence homology (50–60% amino acid identity, including a shared partial von Willebrand factor type D domain), all three RGM molecules are glycosylphosphatidylinositol (GPI)-anchored membrane proteins [19,23-25]. The RGMs were first implicated in both axonal guidance and neural tube closure [23]; more recently, mutations in the gene encoding RGMc have been identified as the leading cause of juvenile hemochromatosis, a condition of iron-overload [26,27]. RGMa and RGMb are expressed prominently in the nervous system [25]. Significant expression of RGMc has been observed in the liver, heart, and skeletal muscle in humans, but is also widely expressed in a host of other tissues, including brain [28].

Here, we further examine the role of the RGMs as co-receptors for BMP2. In addition, because it has recently been shown that BMP12 can signal through the same pathway as BMP2 [29], we have investigated the function of the RGMs in regulating BMP12 signaling as well. Using two separate functional readouts (BMP-responsive reporter gene and endogenous alkaline phosphatase enzyme activity assays), it is shown that siRNA-mediated knockdown of RGMa or RGMc almost completely inhibits BMP2 and BMP12 signaling. While not as dramatic, the specific targeting of RGMb is also shown to significantly impact the BMP induced response. Knockdown of the RGMs failed to affect TGFβ responsiveness in the CAGA-luc reporter assay, demonstrating that the RGMs are important for BMP but not TGFβ responsiveness. Additionally, quantitative binding experiments using surface plasmon resonance allowed us to assess binding affinities between the BMP ligands and RGMa, RGMb, or RGMc. Taken together, these data confirm the role for the RGMs as co-receptors in BMP2 and BMP12 signaling, and that selective knockdown of the RGMs results in dramatic reduction of the BMP responses.

## Results

### siRNA mediated mRNA knockdown

**Figure 1:**
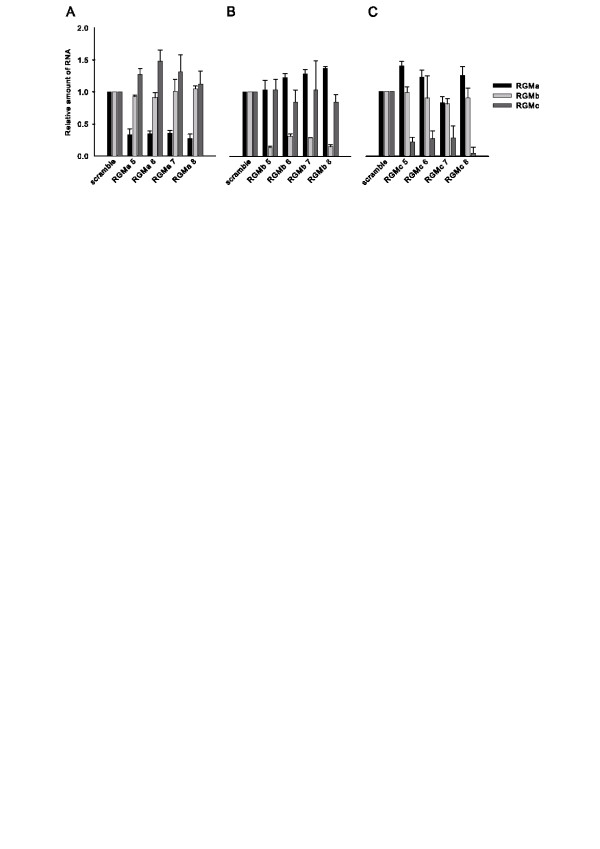
**Relative RNA levels for each RGM after siRNA mediated knockdown**. Real-time quantitative Taqman RT-PCR analysis was performed to test the effectiveness and specificity of RNA target knockdown in cells transfected with siRNA duplexes. A scramble control was tested in addition to four distinct siRNA duplexes that target RGMa (*A*), RGMb (*B*), or RGMc (*C*). Error bars represent the standard deviation (n = 4).

To validate the specificity and efficacy of mRNA knockdown by the siRNA duplexes, we performed real-time quantitative RT-PCR using total RNA extracted from differentially treated cells to define the expression pattern of RGMa, RGMb, and RGMc. For each co-receptor, siRNA duplexes targeting four independent nucleotide sequences were analyzed individually to help control for unintended off-target effects. The results (Figure 1) demonstrate that all 3 RGMs are expressed in C2C12 cells and confirm that each set of siRNA duplexes specifically suppresses mRNA expression of its intended co-receptor, while mRNA levels of the other co-receptors are not suppressed.

### RGM co-receptors are required for significant BMP signaling but not TGFβ signaling

Using the established C2C12 murine myoblast cell line [30,31], targeted knockdown using siRNA duplexes of each RGM co-receptor was performed to examine whether they participate in BMP or TGFβ signaling.

Two independent readouts were used to determine BMP responsiveness, a BMP-inducible luciferase reporter (BRE-luc) and endogenous alkaline phosphatase (ALP) activity. The reporter assay monitors signaling mediated by the BMP-specific Smads [32], while ALP is an enzyme that is upregulated in C2C12 cells in response to BMP but not TGFβ treatment. In addition, TGFβ responsiveness was assessed using the CAGA-luciferase reporter (CAGA-luc), as this has been shown to monitor TGFβ signaling mediated by smad3 [33]. Cells co-transfected with reporter plasmids and siRNA duplexes were treated with BMP2 (100 ng/mL), BMP12 (10 μg/mL), or TGFβ (0.5 ng/mL). The appropriate dose for each inducer was determined by performing a dose-response curve using the relevant reporter. While C2C12 cells are responsive to both BMP2 and BMP12, a much higher dose of the latter is required to induce a response.

**Figure 2:**
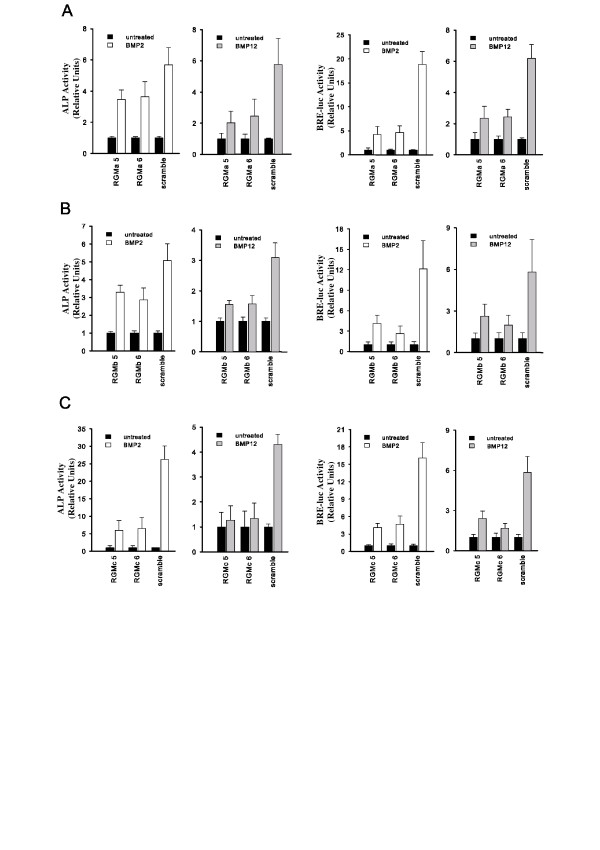
**Cell-based BMP activity assays**. Cells in which RGMa (A), RGMb (B), or RGMc (C) levels were knocked down were treated with either BMP2 or BMP12 and assayed for ALP activity or BRE-luc activity. In each case, data from two effective siRNA duplexes are shown compared to the scramble siRNA sequence. In all cases, activities resulting from BMP2 or BMP12 treatment are normalized to the activity of untreated cells. Error bars represent the standard deviation (n = 4). In all cases, the BMP-induced response from the RGM siRNA-treated cells was significantly lower than for the scramble siRNA-treated cells, as determined by a two-tailed Student's T-test (p-value threshold of 0.05).

**Figure 3:**
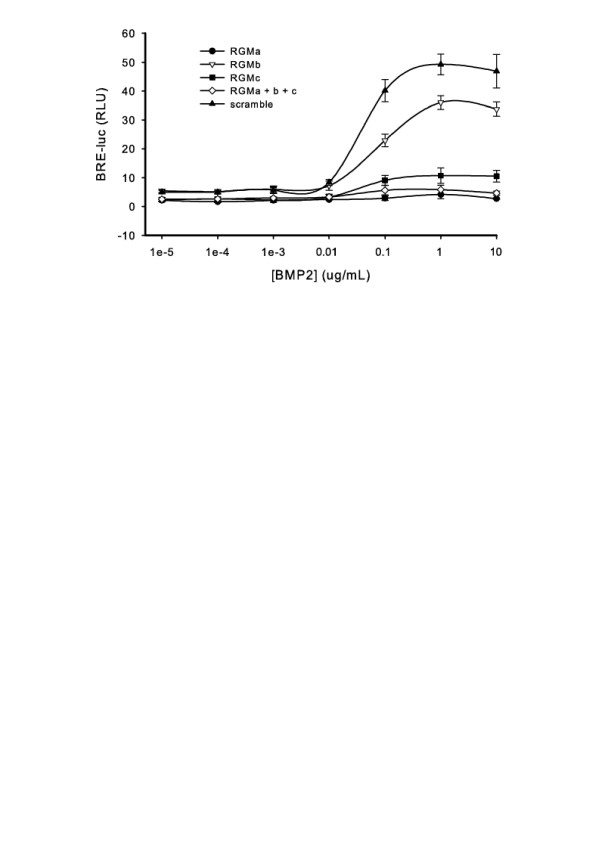
**BMP2 induced BRE-luc activity over a dose range**. RNA knockdown of the indicated target was performed over a range of BMP2 concentrations in C2C12 cells. Error bars represent the standard deviation (n = 4).

**Figure 4:**
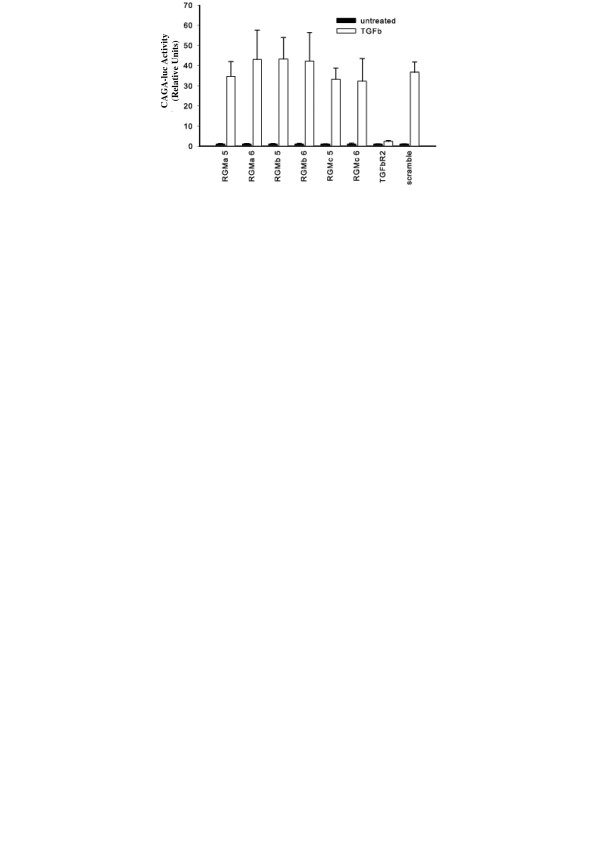
**Cell-based TGFβ activity assay**. Cells in which RGMa, RGMb, or RGMc levels were knocked down were treated with TGFβ and assayed for CAGA-luc activity. Knockdown of TGFβ RII was also performed as a positive control. Error bars represent the standard deviation (n = 4). The TGFβ-induced response from the RGM and scramble siRNA-treated cells showed no significant difference from each other, while the TGFβ R2 siRNA-treated cells showed significantly lower responsiveness, as determined by a two-tailed Student's T-test (p value threshold of 0.05).

Selective knockdown of RGMa, RGMb, or RGMc results in dramatic loss of BMP2 and BMP12 responses in both the BRE-luc and ALP activity assays (Figure 2). To further validate the results shown in Figure 2, we tested the effect of RGM knockdown in the BRE-luc assay over a wide range of BMP2 concentrations. Our results (Figure 3) clearly demonstrate that inhibition of RGM expression blocks reporter activity over a wide range of BMP2 concentrations. Compared to RGMa and RGMc, suppression of RGMb mRNA shows a less dramatic, but still significant, decrease in BMP activity. It should be noted that the suppressive effects of RGM knockdown are not as strong in the ALP assay, likely owing to the fact that the assay takes place several days after the initial siRNA transfection, in which case the RGM levels are likely starting to recover in the cells. TGFβ response, however, was unaffected by the absence of each individual RGM co-receptor (Figure 4). The TGFβ type II receptor (TGFβR2) was knocked down as a positive control, resulting in almost complete loss of cell responsiveness to TGFβ treatment. The lack of a role for the RGMs in the TGFβ pathway is consistent with previous findings in which the RGMs were shown to enhance BMP but not TGFβ signaling [19,20,22].

### RGM co-receptors bind tightly to BMP2 and BMP12

Previous studies have shown qualitatively that RGMa, RGMb, and RGMc interact with BMP proteins [19,20,22]. In order to more quantitatively evaluate the binding interactions between the RGM co-receptors and BMP proteins, binding kinetics and affinities were measured on Biacore sensor chips. Purified RGM co-receptors were individually flowed at a range of concentrations over immobilized BMP2 and BMP12, followed by global analysis of each data set. Biacore analysis demonstrates very tight binding interactions between the RGM co-receptors and the BMP proteins (Table 1) [also see Additional file 1]; all observed interactions displayed k_D_'s in the low nanomolar range. This binding data indicates that the RGM co-receptors likely bind to the BMP ligands on the cell surface, where the RGMs are expressed.

**Table 1:** Kinetic analysis of BMP2 and BMP12 binding interactions with the RGM coreceptors

**BMP2**	**K**_**on **_**(M**^-1^**s**^-1^**)**	**K**_**off **_**(s**^-1^**)**	**K**_**D**_**(nM)**
RGMa	1.48 × 10^5^	3.64 × 10^-4^	2.46
RGMb	4.72 × 10^4^	2.56 × 10^-4^	5.43
RGMc	8.61 × 10^4^	3.63 × 10^-4^	4.22

**BMP12**	**K**_**on **_**(M**^-1^**s**^-1^**)**	**K**_**off **_**(s**^-1^**)**	**K**_**D**_**(nM)**

RGMa	1.27 × 10^5^	1.79 × 10^-4^	1.41
RGMb	5.69 × 10^4^	1.18 × 10^-4^	2.06
RGMc	7.56 × 10^4^	9.22 × 10^-5^	1.22

BIAevaluation software was used to analyze the binding kinetics of BMP2 or BMP12 binding to each RGM co-receptor. In all cases, kinetic parameters were obtained from global analysis of protein binding kinetics [see Additional file 1 for binding curves]

### Transient transfection of RGM co-receptors increases BMP signaling

**Figure 5:**
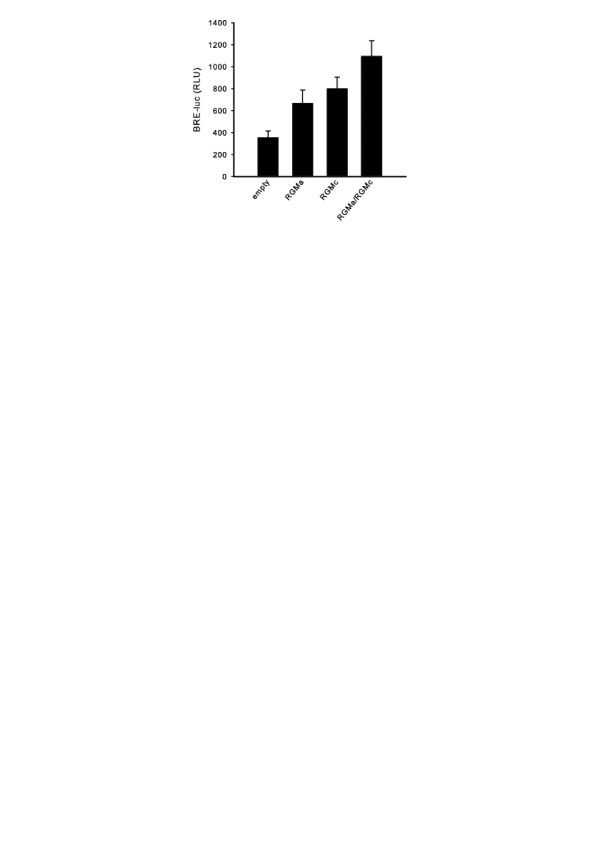
**RGM overexpression in C2C12 cells**. Cells were transiently transfected with the same total amount of either empty vector, RGMa, RGMc, or RGMa/RGMc, followed by BMP2 treatment, and then assayed for BRE-luc activity. Error bars represent the standard deviation (n = 4). Significantly higher BRE-luc activity was observed in cells co-transfected with RGMa/RGMc than in cells transfected in either RGMa or RGMc alone, as determined by a two-tailed Student's T-test (p value threshold of 0.05). Cells transfected without co-receptors (empty vector) showed significantly lower BRE-luc activity than all other treatments.

In the presence of 14 ng/mL BMP2, transient overexpression of RGMa or RGMc increases BMP signaling compared to the empty vector control (Figure 5). Co-transfection of an equivalent total amount of RGMa/RGMc further increased the BMP response in C2C12 cells, demonstrating that BMP2 can more effectively signal using a combination of two distinct RGM co-receptors than using the same amount of a single RGM. RGMa and RGMc were chosen since their knockdown most effectively suppressed BMP signaling in the BMP activity assays.

## Discussion

Originally discovered as cell surface proteins with key developmental roles in the nervous system, recent data have confirmed a role for the RGM family in BMP signaling. Due to the many and varied functions of TGFβ superfamily ligands, multiple levels of regulation are required to tightly control cytokine function. In the BMP signaling pathway, a primary regulatory mechanism is modulation of ligand availability for its receptors. Preventing the BMP/receptor interaction is accomplished by a host of means, including extracellular BMP antagonists, receptor expression, and the presence of BMP pseudoreceptors. In contrast to these inhibitory mechanisms, the recent identification of the RGMs as BMP co-receptors offers a mechanism for enhancement of BMP signaling. Co-receptors, or accessory receptors, have been similarly involved in signaling for several other TGFβ superfamily members: Cripto has been shown to be essential for BMP16/Nodal signaling [18,34], TGFβ type III receptor (also called betaglycan) is involved in TGFβ2 signaling [35], and endoglin has been implicated as an accessory protein in multiple TGFβ superfamily receptor complexes [36].

To better define the roles of the RGMs as co-receptors in BMP signaling, cellular mRNA for each RGM was knocked down via RNAi in cell culture and BMP responsiveness was examined. Additionally, TGFβ responsiveness was examined with the same approach. Interestingly, both BMP activity assays (BRE-luc and ALP enzyme activity) show that loss of either RGMa or RGMc dramatically decreases BMP2 and BMP12 responsiveness. The loss of RGMb has a more modest effect, though still significant. The same results were observed in RGM knockdown experiments that were carried out over a range of BMP2 concentrations (Figure 3). Although it has been shown that BMP12 can signal through the BMP2 pathway [29], the high dose needed to stimulate a BMP12 response in our experimental system suggests that we may be forcing BMP12 to use this pathway. It is possible that lower doses of BMP12 may be able to signal through an as-yet uncharacterized signaling pathway, and the role of the RGMs in this hypothetical pathway are unknown.

To be sure the observed effects were a result of specific RNA degradation, and not off-target effects mediated by the siRNA duplexes, individual experiments were carried out using four unique siRNA nucleotide sequence targets for each RGM co-receptor. Knockdown of RGMa, RGMb, and RGMc was successfully accomplished by each of the four siRNA duplexes targeting the proteins. Additionally, the observed assay results were virtually identical regardless of which of the four sequences was used to suppress mRNA levels (data not shown). As a negative control, ON-TARGET *plus *si *CONTROL *non-targeting pool siRNA duplexes (i.e., "scramble") were transfected in parallel with the RGM-targeting siRNA reagents. Taken together, the data indicate that all 3 coreceptors play roles in potentiating BMP signaling, as knockdown of each significantly reduces BMP response in cell culture.

Previous studies have shown that transient over-expression of RGMa, RGMb, or RGMc significantly increases the sensitivity of BMP-responsive cells in culture [20-22]. Additionally, we observe that transient transfection of each RGM-Fc fusion protein in cell culture shows a repression of BMP activity (data not shown), demonstrating that the soluble forms of these co-receptors are capable of binding to and antagonizing BMP when not bound to the cell membrane.

Given their critical role in BMP signaling, experiments were carried out with the aim of quantifying the interactions between the RGM co-receptors and BMP2 or BMP12. The RGMs have been observed to bind qualitatively to BMP proteins previously, with a published equilibrium dissociation rate constant (k_D_) only for the RGMb:BMP2 interaction [19]. We therefore sought to quantitatively analyze the binding of BMP2 and BMP12 to each RGM protein using a Biacore platform. The binding results show that all three RGM proteins bind with very high affinity (equilibrium dissociation constants, k_D_, in the low nanomolar range) to both BMP2 and BMP12. These tight affinities further confirm the observations that each RGM protein is capable of binding directly to the BMP ligands. This suggests that in their native GPI-bound form on the cell membrane, the RGM co-receptors are able to directly bind the BMP ligands; it is possible that this direct binding may lead to enhanced cytokine recruitment to the type I/II receptor complexes.

Perhaps the most interesting data from our studies is evidence that the RGM co-receptors are not able to compensate functionally for one another. All 3 RGMs are expressed in our cell system, C2C12; if the RGMs have redundant activities, knocking down levels of one co-receptor would not lead to a significant phenotypic result due to the presence of the other two. In such a case, knockdown of all three receptors would be needed before the signaling pathways are affected. However, our data show that reduced expression of a single RGM protein suppresses BMP activity in cells. The collective data suggest a mechanism in which the signaling BMP receptor complex requires two distinct RGM co-receptors for efficient signaling, though it is unclear whether they exist as a heterodimer or two distinct proteins. In this model, the possibilities are RGMa/RGMb, RGMa/RGMc, or RGMb/RGMc. In our cell system, RGMa/RGMc is the most effective combination, as suppression of either one significantly blocks BMP activity. Since knockdown of RGMb has the least significant effect, this suggests that complexes involving RGMb form less effective signaling complexes. Previous studies have shown that the RGM proteins have the ability to participate in BMP-receptor complexes. Thus, it appears that the presence of two RGM proteins enhances BMP ligand recruitment to the membrane-bound receptors, and helps generate an active signaling complex. Our data indicate that RGMa and RGMc form the most potent co-receptor pair in the context of our *in vitro *cell system. In accord with previous results, we observe no involvement of the RGM proteins in TGFβ signaling.

According to our proposed mechanism, in which the RGMa/RGMc combination of co-receptors is most potent at facilitating BMP signaling, cells that overexpress this combination should show improved BMP signaling versus cells that overexpress RGMa or RGMc individually. Experiments in which cells are transiently transfected with the same total amount of empty vector, RGMa, RGMc, or a RGMa/RGMc combination show a statistically significant (p value < 0.05, n = 4) increase in BMP activity (BRE-luc readout) when the RGMa/RGMc combination of co-receptors is expressed versus RGMa or RGMc individually. Overexpression of RGMa or RGMc individually also increase BMP signaling versus empty vector controls, consistent with previously published data [19,20,22].

In addition to our cell-based assay data, several lines of evidence support our proposed mechanism. Analysis of endogenous RNA expression levels in a variety of BMP responsive cell lines (C2C12, C3H10 T 1/2, 3T3L1, and MLB13MYC Clone 14) shows that RGMa and RGMb RNA is abundant in each cell line, while RGMc RNA is appreciably expressed in C2C12 and MLB13MYC Clone 14 (data not shown). This expression data supports the hypothesis that two of the RGM co-receptors are needed for effective BMP signaling, since all tested BMP-responsive cell lines express at least two of the RGM proteins. Also, there is evidence that the RGM proteins do not functionally compensate for one another. It has been shown that mutations to the gene encoding RGMc are a leading cause of juvenile hemochromatosis, presumably due to loss of functional RGMc protein. The fact that RGMa and RGMb do not rescue this disease phenotype (when caused by mutated RGMc) suggests that they are not functionally redundant with RGMc protein. While all these results support our proposed mechanism, further experiments are required to more fully elucidate it. Alternatively, other explanations are possible. For example, the RGMs may act as monomeric coreceptors that can operate by different signaling routes; these pathways may be functionally distinct in certain BMP-induced activities (such as those measured in our experiments) but not in others. Additional studies are required to fully understand the mechanisms through which the RGMs regulate BMP signaling.

## Conclusion

Our results build upon previous studies that have demonstrated that each RGM protein serves as a BMP co-receptor. It is clear that not only does the presence of these co-receptors potentiate BMP signaling, but also that their absence diminishes BMP responsiveness *in vitro*. Thus, the RGM co-receptors are necessary for significant signaling through the BMP, but not the TGFβ, pathway. Based on our results, a pair of RGM co-receptors is minimally required to form an effective signaling complex, though the details of this process are still unclear.

## Methods

### Reagents and Proteins

Human BMP2 was produced and purified as previously described [37]. Human BMP12 was produced in *E.coli *and purified through a series of SP-Sepharose cation exchange column steps, with final purification achieved by size exclusion chromatography on two tandem TOSOH G3000SWL columns. The BMP12 dimer peak from the size exclusion column was dialyzed to 0.1% TFA followed by concentration on a ThermoSavant SPD SpeedVac. TGFβ-1 protein was purchased from R&D Systems.

### Receptor-Fc production and purification

RGMa-Fc, RGMb-Fc, and RGMc-Fc were produced in FreeStyle 293-F cells (Invitrogen) transiently transfected with expression plasmids encoding the ectodomain of each RGM fused to the Fc portion of human immunoglobulin (IgG) to generate soluble fusion protein. Transfections were performed with 293Fectin (Invitrogen) in serum-free medium according to the manufacturer's instructions. For each Fc-fusion protein, ~40 mL of conditioned medium was centrifuged, filtered, and diluted 4-fold in 20 mM sodium phosphate (pH 7.0) before one-step purification on a Protein G affinity column (HiTrap Protein G HP, Amersham Biosciences). After elution in 0.1 M glycine (pH 2.7) and addition of Tris-HCl (pH 9.0) to neutralize pH, the protein was concentrated and the buffer exchanged using a Centricon centrifugal filter device (Millipore). Purified RGM-Fc's were analyzed using SDS-PAGE; to confirm purity, gels were either blue stained or transferred to a nitrocellulose membrane and Western blotted using α-human IgG polyclonal antibodies.

### BMP and TGFβ activity assays in C2C12 cells

C2C12 cells [30,31] were transiently transfected in 6-well plates using Lipofectamine 2000 (Invitrogen) with a BMP responsive firefly luciferase reporter (BRE-luc [32]) or a TGFβ responsive firefly luciferase reporter (CAGA-luc [33]) with pRL-TK Renilla luciferase vector to control for transfection efficiency, in combination with the si *GENOME *ON-TARGET *plus *siRNA duplexes (Dharmacon) targeting a specific co-receptor, receptor (TGFβR2), or scramble sequence. Dharmacon catalog numbers for these reagents are LU-055474-00-0002 (RGMa), LU-055534-00-0002 (RGMb), LU-055494-00-0002 (RGMc), LU-040618-00-0002 (TGFβR2), and D-001210-01-05 (scramble sequence). After 24 hours, the cells were trypsinized, harvested, and either reseeded into 96-well plates or used for RNA extraction (see below). Reseeded cells were treated with purified ligand (100 ng/mL BMP2, 10 μg/mL BMP12, or 0.5 ng/mL TGFβ) in DMEM supplemented with 1% FBS and assayed for either luciferase activity (after 24 hours) or alkaline phosphatase (ALP) activity (after 4 days). Luciferase activity was determined after cell lysis using the Dual-Glo Luciferase Assay System (Promega), and all experiments were performed in quadruplicate wells. In all cases, firefly luciferase levels were normalized to the corresponding amount of Renilla luciferase in a given well, yielding the final values in relative luciferase units. ALP activity assays were performed as described previously [38]. Briefly, following cell wash and lysis, ALP activity of the cell lysates was tested in assay buffer (0.1 M glycine, 8 mM MgCl_2_, and 0.1% Triton X-100) using 10 mM p-nitrophenyl phosphate as a substrate at 37°C. ALP activity was normalized to DNA levels in each well to account for potential differences in cell number.

### Quantitative Real-Time PCR analysis of Receptor Expression

Messenger RNA was extracted and purified from the transiently transfected C2C12 cells after 24 hours using the RNeasy mini kit (Qiagen, Valencia, CA), including an on-column DNAse I treatment step to digest any contaminating genomic DNA. Real-time quantitative RT-PCR was performed in a one-step procedure combining cDNA synthesis with PCR amplification on an ABI Prism 7900 HT Fast Real-Time PCR system (Applied Biosystems). Sequence specific primers and FAM-labelled probes used for individual genes were also obtained from Applied Biosystems, catalog numbers Mm00624998_m1 (RGMa), Mm00724273_m1 (RGMb) and Mm01265683_m1 (RGMc). The RT step involved incubation at 48°C for 30 min, followed by the PCR cycling conditions (initial denaturation of 95°C for 10 min, followed by 40 cycles of 95°C for 15 seconds and 60°C for 1 minute) in 96-well plates. For each reaction, the cycle threshold (C_t_) was determined as the cycle number at which a threshold fluorescence value (set in the exponential phase of the amplification curves) was reached. In order to measure reaction efficiency, a standard curve was generated using 100, 20, 4, 0.8, and 0.16 ng of total starting RNA. In all cases, gene expression levels were normalized to GAPDH expression levels in order to assess changes in relative expression.

### Binding analysis using surface plasmon resonance (Biacore)

Binding experiments and kinetics analyses were performed using the BIAcore 3000 (Biacore), the basic principles of which are well documented [39,40]. Purified recombinant human BMP2 or BMP12 were prepared in immobilization buffer (10 mM Acetate, pH 4.5) at a concentration of 10 ug/mL and were immobilized on sensor chips (CM5, research grade, Biacore) by the amine coupling method at a flow rate of 10 ul/min at 25°C. The immobilization levels for BMP2 and BMP12 were 350 and 1000 RUs, respectively. For binding analysis, soluble receptor (RGMa, RGMb, or RGMc) was injected over a range of concentrations between 0.1 nM to 100 nM at 25°C at a flow rate of 30 uL/min for 500 s. The resulting data from multiple experiments were globally analyzed using BIAevaluation software version 3.2 (Biacore) to determine kinetic parameters, association rate constant (k_a_), and dissociation rate constant (k_d_). Degassed HBS-EP (0.01 M HEPES, 0.15 M NaCl, 3 mM EDTA, and 0.005% Nonidet P-40, pH 7.4) was used as running buffer and for sample dilution. Surface regeneration after ligand binding was efficiently accomplished by injection of 100 mM HCl (20 uL), as determined by preliminary experiments. In all cases, double referencing [41] was performed to correct for any non-specific binding or buffer related artifacts.

### Overexpression of RGM proteins in C2C12 cells

C2C12 cells were transiently transfected in 96-well plates with an empty vector control, RGMa, RGMc, or a combination of 1:1 RGMa/RGMc. In all cases, the same total amount of expression plasmid was transfected into each well. Simultaneous co-transfection of the BRE-luc and pRL-TK Renilla reporters was performed as above. In initial experiments, Cells were treated with 100 ng/ml of BMP2 for 24 hours and BRE-luc activity was assayed as described above. Subsequent experiments found that a lower dose of BMP2 (14 ng/ml) was more effective.

## Abbreviations

TGFβ, transforming growth factor β; BMP, bone morphogenetic protein; GDF, growth and differentiation factor; RGM, repulsive guidance molecule; GPI, glycosylphosphatidylinositol; TFA, trifluoroacetic acid; Tris-HCl, Tris (hydroxymethyl) aminomethane hydrochloride; SDS-PAGE, sodium dodecyl sulfate polyacrylamide gel electrophoresis; siRNA, small interfering RNA; DMEM, Dulbecco's modified Eagle's medium; ALP, alkaline phosphatase; RT-PCR, reverse-transcriptase polymerase chain reaction; GAPDH, glyceraldehyde-3-phosphate dehydrogenase; RU, resonance units.

## Competing interests

The author(s) declare that they have no competing interests.

## Authors' contributions

PJH, JMW and GB designed the study and analyzed the results. PJH and RD performed the experiments. PJH and GB wrote the manuscript. All authors read and approved the final manuscript.

## Supplementary Material

Additional file 1 **Biacore biosensor binding data**. Sensorgrams from Biacore kinetic analysis of BMP2 and BMP12 binding to the RGM co-receptors. A concentration series of receptor is shown in color, with the kinetic fits (from BIAevaluation software) overlaid in black. Binding curves are shown for the interaction of RGMa/BMP2 (*A*), RGMa/BMP12 (*B*), RGMb/BMP2 (*C*), RGMb/BMP12 (*D*), RGMc/BMP2 (*E*), and RGMc/BMP12 (*F*). Click here for file
