# Supplementary figures and images for: Role of RGM coreceptors in bone morphogenetic protein signaling
Source: J Mol Signal. 2007 Jul 5;2:4. doi: 10.1186/1750-2187-2-4 (PMC1933414; doi:10.1186/1750-2187-2-4)

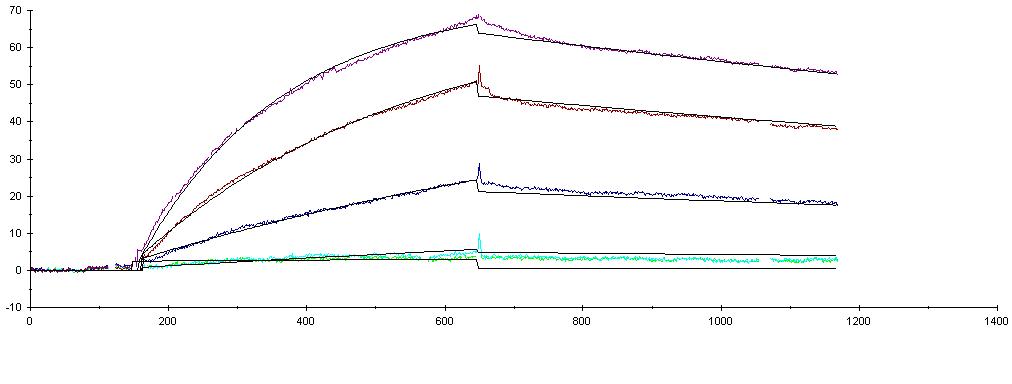

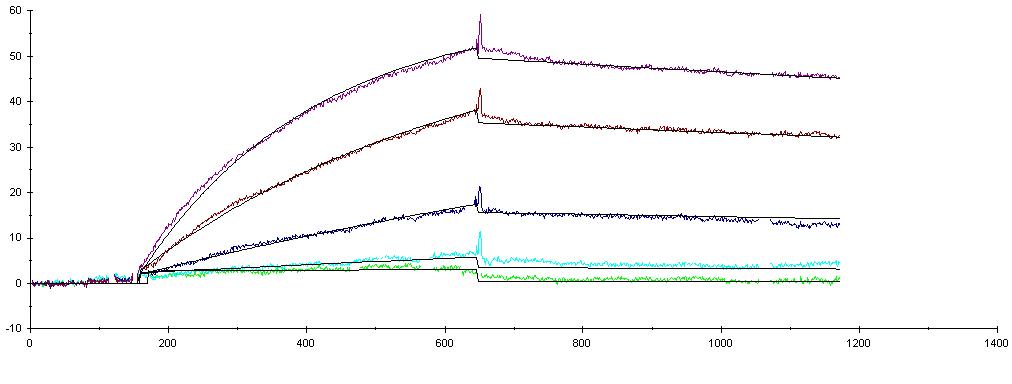

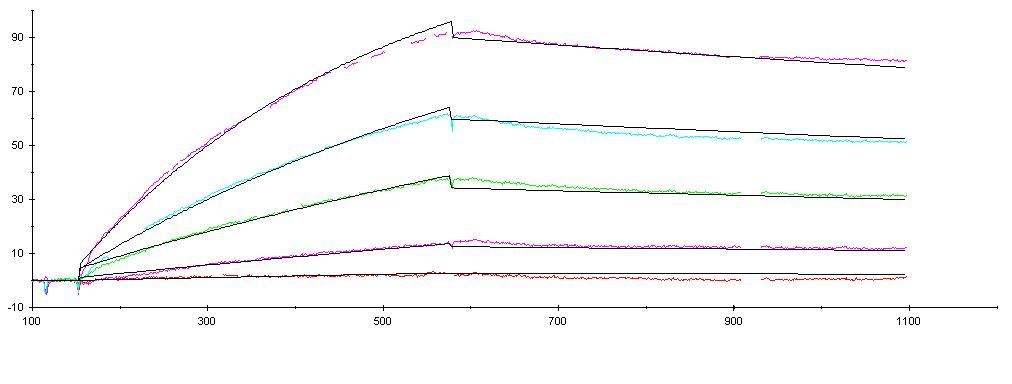


A.

B.

C.

D.


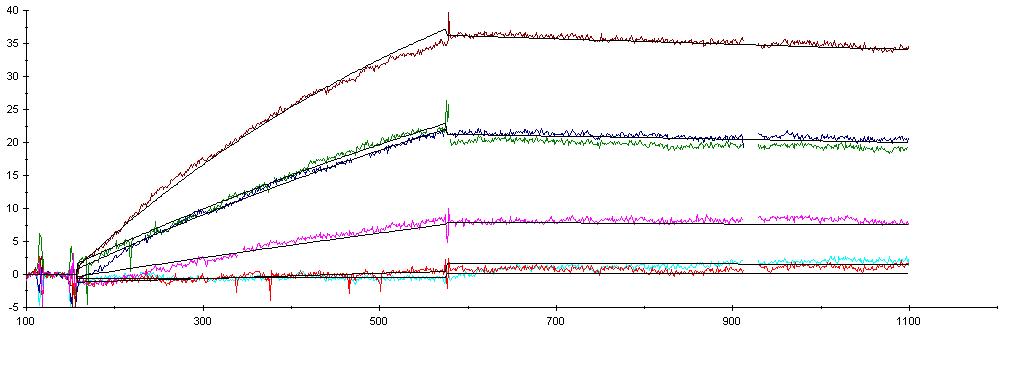


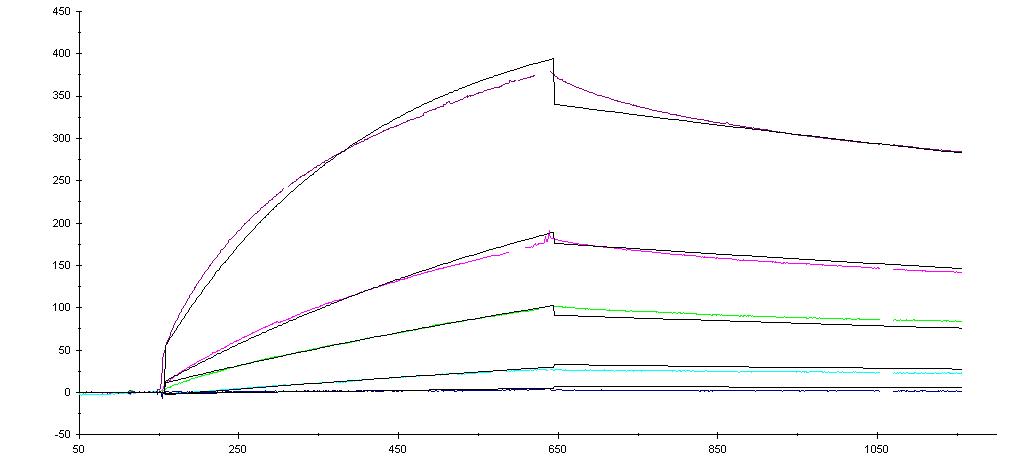

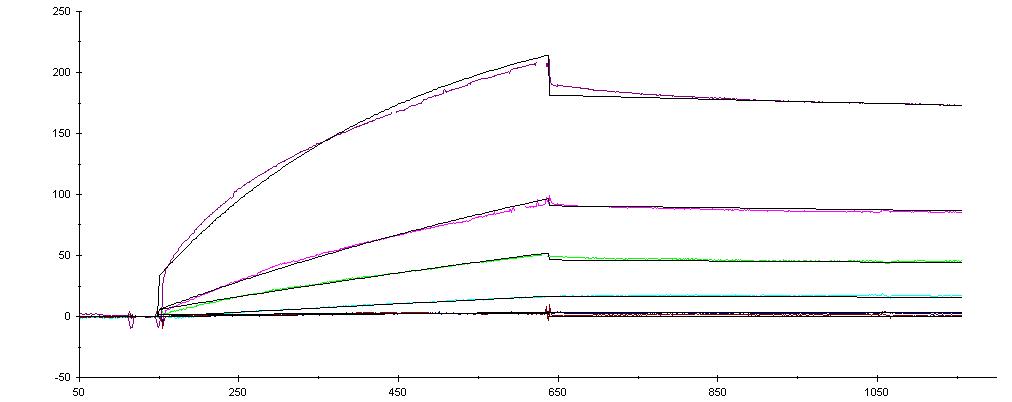


E.

F.

Supplement: Additional file 1 — Biacore biosensor binding data. Sensorgrams from Biacore kinetic analysis of BMP2 and BMP12 binding to the RGM co-receptors. A concentration series of receptor is shown in color, with the kinetic fits (from BIAevaluation software) overlaid in black. Binding curves are shown for the interaction of RGMa/BMP2 (A), RGMa/BMP12 (B), RGMb/BMP2 (C), RGMb/BMP12 (D), RGMc/BMP2 (E), and RGMc/BMP12 (F). [file 1750-2187-2-4-S1.doc]
